# Spiking neural networks for predictive and explainable modelling of multimodal streaming data with a case study on financial time series and online news

**DOI:** 10.1038/s41598-023-42605-0

**Published:** 2023-10-26

**Authors:** Iman AbouHassan, Nikola K. Kasabov, Vinayak Jagtap, Parag Kulkarni

**Affiliations:** 1 Technical University of Sofia, Sofia, Bulgaria; 2 Central Bank of Lebanon, Beirut, Lebanon; 3 KEDRI, SECMS, Auckland University of Technology, Auckland, New Zealand; 4 Ulster University, Belfast, UK; 5 IICT, Bulgarian Academy of Sciences, Sofia, Bulgaria; 6 College of Engineering, Pune, India; 7 Tokyo International University, Tokyo, Japan

**Keywords:** Information technology, Computational science, Engineering, Mathematics and computing

## Abstract

In a first study, this paper argues and demonstrates that spiking neural networks (SNN) can be successfully used for predictive and explainable modelling of multimodal streaming data. The paper proposes a new method, where both time series and on-line news are integrated as numerical streaming data in the same time domain and then used to train incrementally a SNN model. The connectivity and the spiking activity of the SNN are then analyzed through clustering and dynamic graph extraction to reveal on-line interaction between all input variables in regard to the predicted one. The paper answers the main research question of how to understand the dynamic interaction of time series and on-line news through their integrative modelling. It offers a new method to evaluate the efficiency of using on-line news on the predictive modelling of time series. Results on financial stock time series and online news are presented. In contrast to traditional machine learning techniques, the method reveals the dynamic interaction between stock variables and news and their dynamic impact on model accuracy when compared to models that do not use news information. Along with the used financial data, the method is applicable to a wide range of other multimodal time series and news data, such as economic, medical, environmental and social. The proposed method, being based on SNN, promotes the use of massively parallel and low energy neuromorphic hardware for multivariate on-line data modelling.

## Introduction

### Integrated modelling of multimodal streaming data

Integrated modelling of multimodal streaming data is an open problem^1–5^. Methods for integrating multimodal data into one machine learning model have been developed across domain areas, such as fault detection and diagnostics in the context of sparse multimodal data and expert knowledge assistance^6–8^. In^9^, application of integrated approach is demonstrated for hydro generators. The published models are mainly dealing with vector-based data rather than time series and usually incorporate static rule-based knowledge into a computational model, such as a neural network, before the model is trained on data, rather than integrating dynamically both time series and on-line news/text information^6^.

The problem of integrating time series with on-line news and text information has been acknowledged^10^, with only few studies suggesting effective methods. For example, a deep learning approach used text data to reduce errors in the prediction of taxi demand^11^. In^12^, text is used for financial data analysis using classical statistical machine learning. In^13^, Hong Kong market data was used to find a correlation between stock values and financial news. In^14^, a combination of text information with a statistical feature in Financial LDA is proposed. In^15^, stock market data are associated with financial data for classification using time series methods like DTW and SAX. In^16^, 5-year stock and news data were used to find the association between them with statistical analysis. In^17^, it was demonstrated that sentimental polarities matter in stock market forecasting. In^18^, news polarity and stocks were interlinked, and sentimental analysis helped to find some associations between stock data and financial news. In^19^, it is demonstrated that volatility movements are more predictable than asset price movements when using financial news. In^20^, it was demonstrated that there is improvement in forecasting by acquiring the right contextual information for financial data. A strong correlation between stock data and acquired financial text was found in^21^. Prediction of stock market direction using financial news was studied in^22,23^. It was found that there is an impact of news data on financial data to predict if the market will rise or fall. Other machine learning methods, such as SVM and PSO were also tried for sentiment analysis of financial data to show improvement compared with the use of deep learning mechanisms.

The above researches confirmed that on the one hand the integration of time series and news information may improve the quality of time series and on the other hand they raise the issue of lacking efficient methods for the task, as neither of the above methods were able to learn integrated time series data in an on-line, incremental and adaptive mode and most importantly, to extract meaningful dynamic patterns of interaction of the used variables for a better understanding of the modelled processes.

A challenge is what multimodal streaming data and information to use and how to encode and combine all this information into one model for a better online predictive and explainable learning. News information can include textual data, reflecting on industry performance, economic conditions, and political events. For example, news has a significant impact on stock market sentiment and prices^24,25^. News information can be obtained from various business websites that analyze financial data, such as stock market, commodity prices etc.

Traditional machine learning techniques have already been used to extract and classify news from trusted sources^11–27^. Despite the work done, there are limitations in these methods and there are still open questions, that are addressed in this paper:

- How to encode online news for a particular type of time series and how to evaluate their impact?
- How to integrate time series and online news incrementally in a predictive model and how to evaluate the impact of the news on the model outcome?
- How to reveal inherent and interpretable patterns of dynamic interaction between time series variables and online news, which patterns may change over time?

### Why use spiking neural networks (SNN)?

To address the above research questions, we base our methods on the third generation of neural networks, spiking neural networks (SNN), because SNN have the ability to capture dynamic spatio-temporal patterns from multi-input streaming data^28–30^ and some SNN models allow for dynamic spatio-temporal patterns to be interpreted and explained^31–33^.

Learning in SNN is inspired by the human brain as the brain is capable of on-line integration of different sources of temporal and textual information over time. Integrating information from different sources is crucial for a better decision making and event prediction by humans, crucial for their survival. The challenge addressed in this paper is how to apply SNN to develop models that integrate multiple time series and other types of related online information, for a better predictive modelling and for a better understanding of the dynamic relationship between the data and other relevant information sources.

Various spiking neuron models have been proposed in the literature, including the popular Leaky Integrate and Fire neuron (LIF), spike response neuron, probabilistic spiking neuron^31–34^, etc., along with learning algorithms for unsupervised and supervised learning, one of them being the spike-time dependent plasticity (SDTP) algorithm^35^. Various architectures of SNN have been proposed including: deep SNN^36^; evolving SNN (eSNN)^37^; dynamic evolving SNN (deSNN)^38^; spike-pattern association neurons and networks (SPAN)^39^, NeuCube^32^.

SNN have been used as a computational paradigm for the development of neuromorphic hardware as massively parallel computational architectures that consume thousands of times less energy^40–42^. Different types of SNN offer different specific characteristics, that make them more or less suitable for the task in hand. So which method to use? Our proposed model uses NeuCube as a suitable, brain inspired SNN architecture as explained in the paper.

The main contributions of the paper are: (1) a method for feature extraction and encoding of online news and integrating them with time series data; (2) a method, based on SNN, for an integrated predictive modelling of multimodal time series; (3) a method for the discovery of explainable dynamic patterns of interaction between multimodal time series variables; (4) predictive and explainable modelling of stock price time series, integrated with online news.

## Results

### Predictive modelling and understanding of multiple time series integrated with online news

This section presents results on integrated modelling of multiple time series and on-line news using the methodology described in the method section. While the proposed method can be applied on any various time series data and related on-line news, here it is applied on stock indexes and on-line news. The results demonstrate the benefit of using news when predicting one of the time series.

#### Multiple time series data

Here we show results on a selected stock market dataset that contains 7K+ stock records for daily prices with multiple variables {https://www.kaggle.com/itoeiji/deep-reinforcement-learning-on-stock-data}. The data records daily closing adjusted prices of eight stock indices. The observations are daily time series that span from January 3rd, 2017 to June 30th, 2017, with 129 observations total for each stock index. Weekends are removed, and missing values, due to holidays, are replaced with the prices immediately preceding them. The dataset comprises the following stock indices: Wipro Limited [WIT], Microsoft Corporation [MSFT], Lennar Corporation [LEN], International Business Machines [IBM], Adamis Pharmaceuticals Corporation [ADMP], Alphabet Inc. [GOOG], Reliance Industries Ltd. [RIL], and Tata Consultancy Services [TCS]. The raw data sample consists of ordered daily sequences of adjusted closing prices. Descriptive statistics for the input variables along with their evolution in time are shown in Supplementary Table S1 and Supplementary Fig. S1, respectively. The output variable here represents the following day's closing price of the Indian Wipro Limited multinational corporation [WIT] (Suppl. Fig. S2).

#### Data preparation

Using a sliding window approach, the original dataset is segmented into 70 window samples, each of size 60 with a sliding step of one, resulting in 4200 data points used to feed, learn, and test the model. The sliding window collects historical time series data in order to train the model and forecast the next day's closing price of the stock. When the actual output results are available, they can be incrementally added to the SNN model for further training. The news variable, which is encoded on a day-based value between 1 (strong positive news related to the output variable and − 1 (strong negative news), is associated with the stocks.

The real input data is encoded from continuous values to discrete sequences of spikes that represent changes in the data at times. The Threshold-based Representation (TR) encoding method, with a spike threshold of e.g., 0.5, is used to discretize the continuous streams of data into spikes. The spike information representation simplifies the input data by focusing on the changes in the data over time rather than using their real values in the followed modelling procedure, that aims at learning dynamic patterns of these changes that relate and trigger certain outcomes. This is a principle adopted from how the brain processes continuous value sensory information.

#### Results of computational modelling using the proposed method from the Methods section

**Figure 1:**
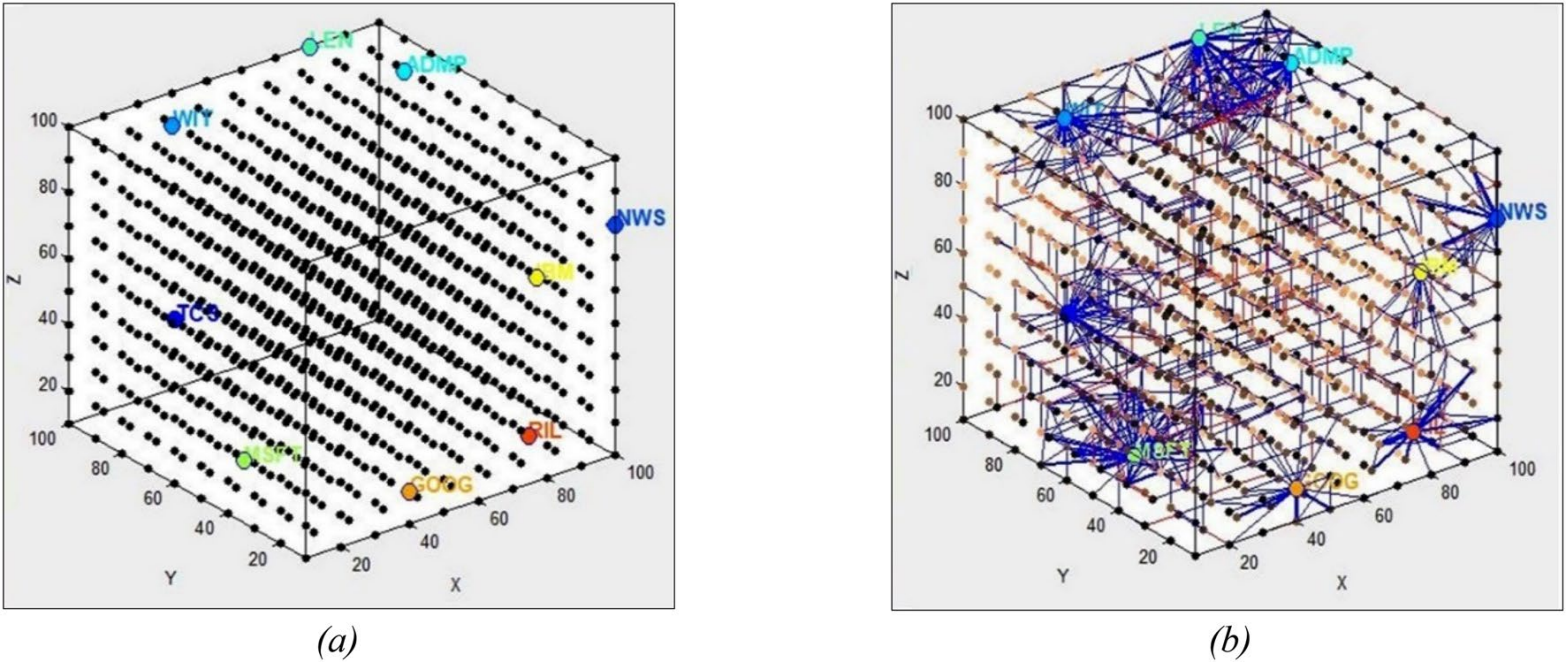
(**a**) Results of mapping time series variables including online news variable (NWS) into a 3D SNN (the size of the SNN is scalable); (**b**) resulted connectivity of a trained SNN model on the 8 stock time series and on-line news (NWS).

All input variables, including the news variable, are mapped into the 3D SNN from a NeuCube model, based on their temporal similarity in the training data measured as time series similarity, so that similar input features are allocated to closer spiking neurons (input neurons) in the 3D space of the SNN, based on a graph matching algorithm to automatically generate a scalable 3D SNN, e.g., 1000 (10 × 10 × 10) neurons in a 3D reservoir (Fig. 1a)^43^. This algorithm allocates the spatial coordinates of the input neurons representing the input variables based on the temporal similarity in the training data measured as time series similarity, so that similar input features are allocated to closer spiking neurons (input neurons) in the 3D space. The connections between the spiking neurons in the SNN are initialized using the small world connectivity rule, that creates probabilistically positive or negative connections of small values based on the distance between the neurons, the closer the neurons in the 3D space, the higher the probability to connect.

The encoded input data into spike trains are mapped into spatially located neurons in a 3D SNN, with an average spike rate of 0.22 at a time length of 5 for this particular data. The encoded spike sequences are continuously fed into the SNN reservoir in the order they were encoded. In this experiment 50% of the time series data is used for training and the future 50%stock values is used for incremental learning and testing the model. The used SNN and deSNN regressor parameter values are shown in a software panel in Supplementary Fig. S2.

The SNN is first *trained incrementally* in an unsupervised mode using the STDP rule on the samples S of the integrated time series and online news data. A resulting trained SNNcube on exemplar data is shown in Fig. 1b. The STDP learning rule considers the time of spiking between two connected neurons, so that consecutive changes in data from one time to another across all input variables are learned. STDP is expressed in terms of STDP learning window $({\tau }_{pre}-{\tau }_{post})$ in which the difference between the time of a pre-synaptic spike and the time of a post-synaptic spike will determine the synaptic weight (see Eq. 7).

The connections of a resulted SNN model after unsupervised learning are shown in Fig. 1b.

**Figure 2:**
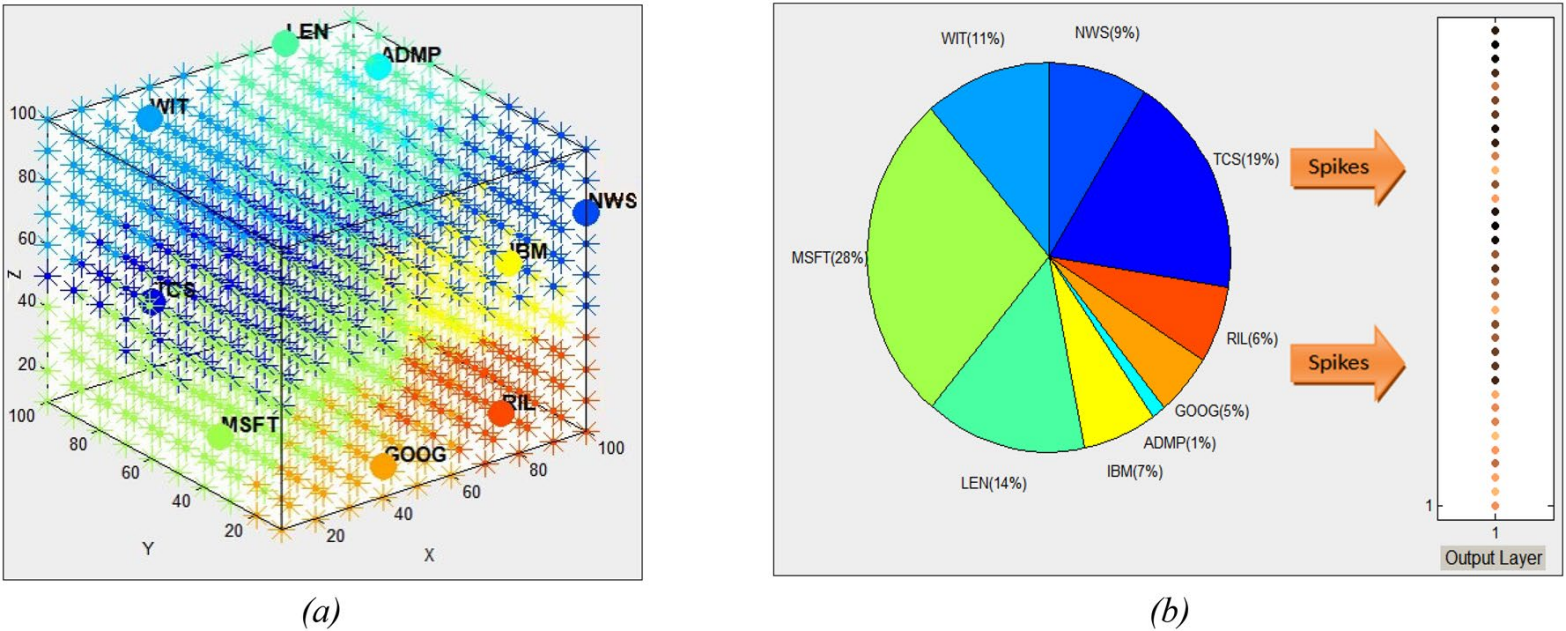
Results of the analysis of the trained model on stock indexes and on-line news: (**a**) clustered neurons according to their maximum connection weight with an input neuron (input variables); (**b**) percentage of neurons with maximum connection weights to each of the input neurons represents the impact of the input variables on the model functionality and can be used for feature selection or predictive marker discovery. This ranges from 1% for ADMP to 28% for the Microsoft index, while a news indicator NWS accounts for 9%.

For a better *understanding* of the dynamic interaction in time between different input variables, several analysis techniques are applied on the SNN connectivity and spiking activity. First, neurons in the SNN are clustered around the input neurons (representing the input variables) according to their strongest connection weights with an input variable (similar to a membership function in fuzzy clustering) (Fig. 2a). The percentage of neurons with maximum connection weights to each of the input neurons represents the impact of the input variables on the model functionality and can be used for feature selection or predictive marker discovery (Fig. 2b). This percentage ranges from 1 to 28% for the Microsoft index in the example, while a new indicator NWS accounts for 9%. Similar graphs of a SNN model for predicting the same index without using news information are presented in the Supplementary materials Fig. S3.

**Figure 3:**
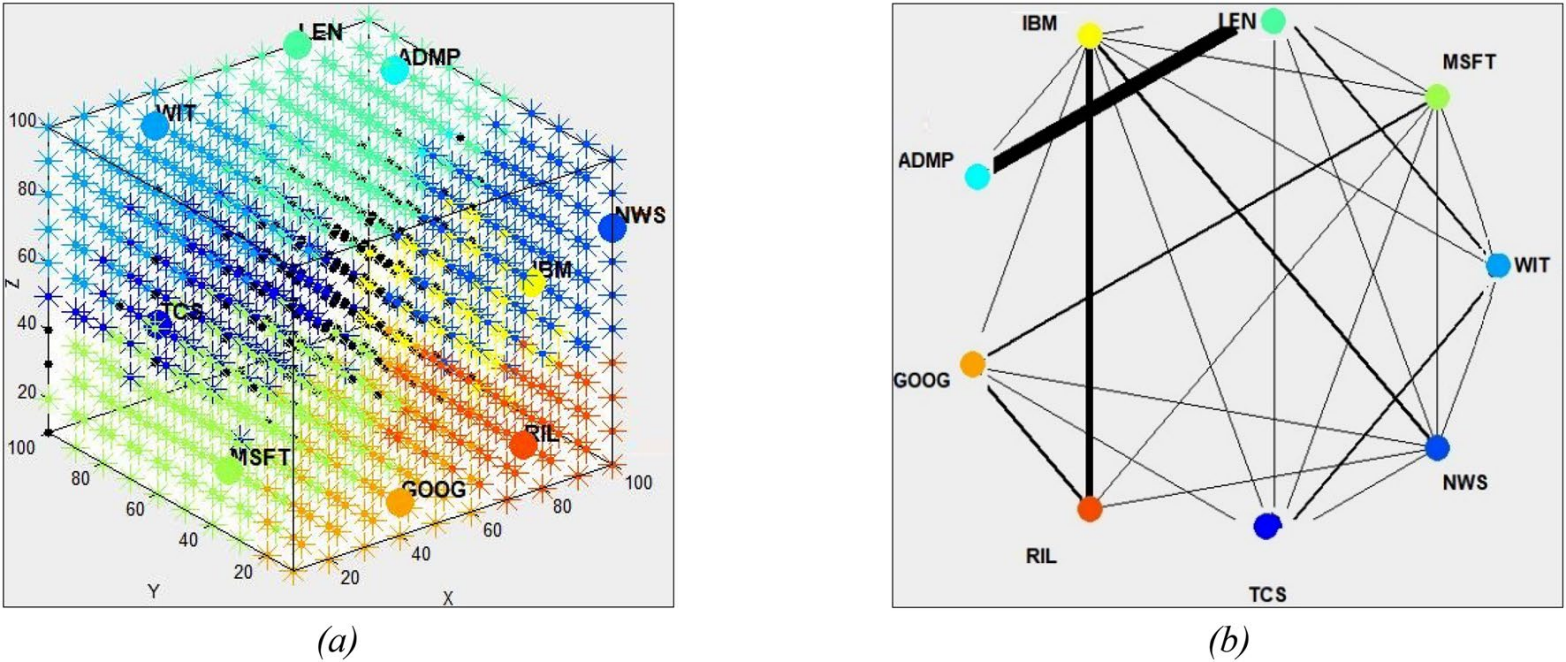
Results of the analysis of the trained SNN model on stock time series and on-line news (NWS): (**a**) neurons in the SNN model are clustered according to the maximum number of spikes received from an input neuron, which shows the dynamics of the interaction between the neurons; (**b**) this is represented as a dynamic graph, where the thickness of the arcs shows the intensity of spike exchange between the corresponding input neurons (a feature interaction network).

Neurons in the SNNcube are also clustered according to the maximum number of spikes received from an input neuron, which shows the dynamics of the interaction between the neurons (Fig. 3a). This is represented as a dynamic graph (Fig. 3b) where the thickness of the arcs corresponds to the intensity of spike exchange between the corresponding input neurons (a feature interaction network).

Figure 3b indicates the dynamic relationships between the used stock variables and the online news variable. It shows that the Lennar Corporation [LEN] stock and the Adamis Pharmaceuticals Corporation [ADMP] stock is relatively interdependent, so are the IBM and the Indian Reliance Industries [RIL] stocks.

After a SNNcube model is trained, an output regression module is trained using a SNN regressor deSNN, which is a computationally efficient model that prioritizes the first spike arriving at the output neurons^38^. The supervised training of the output regressor is performed using predefined output values of the dependent variable under study that are associated with the training samples. Based on the rank-order (RO) learning rule, the deSNN Mod parameter is set to 0.8. This is the modulation factor used to calculate the initial weight of every new connection formed from the SNN cube to an output neuron representing the desired output value. In order to reflect the following spikes on the same synapse, these connection weights are further adjusted using a drift parameter set here to 0.005.

**Figure 4:**
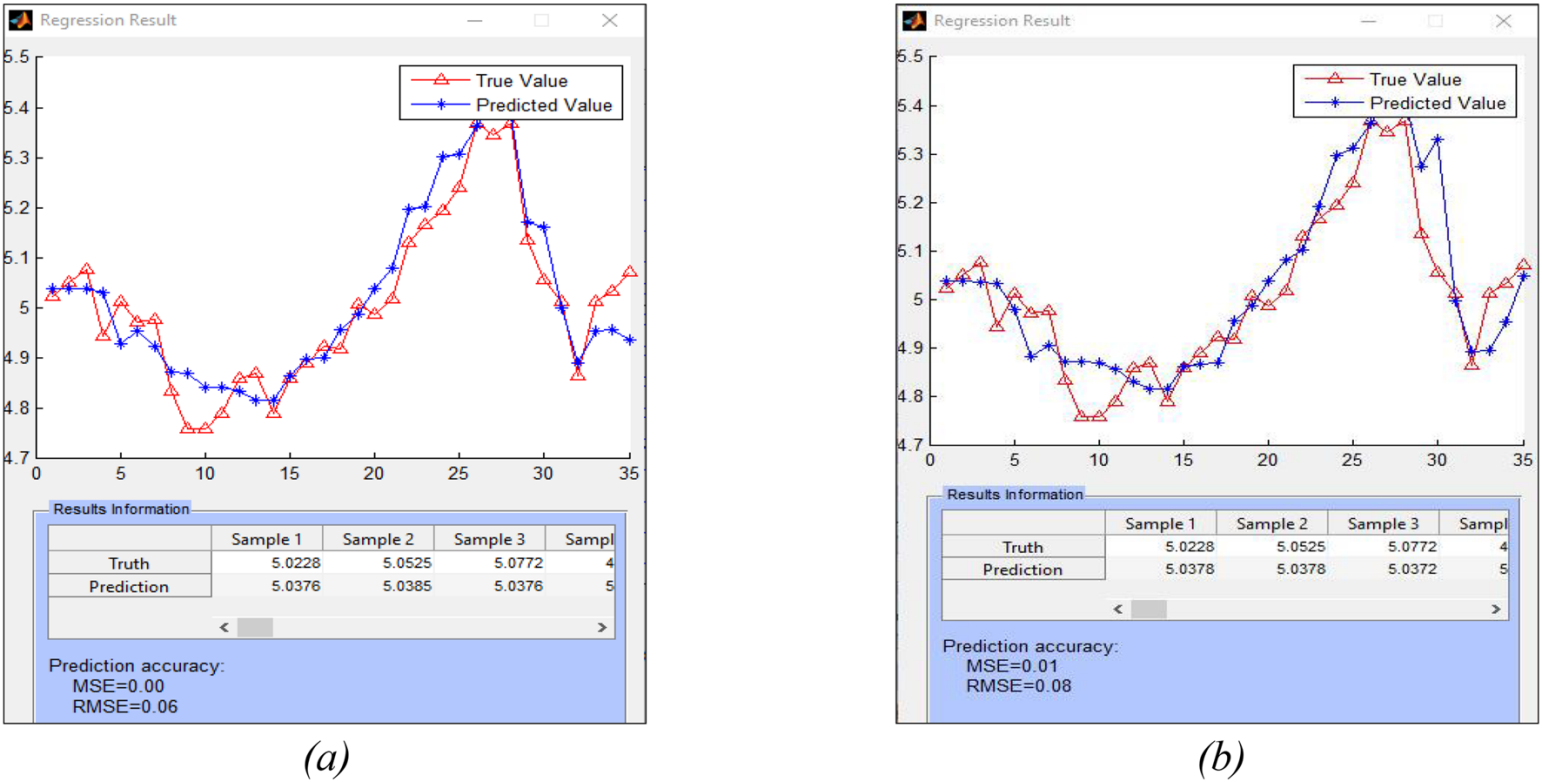
The resulted accuracy of incremental learning and prediction of the WIT stock value 1 day ahead, using previous 60 days values of the 8 stock variables and the online news, results in a smaller predictive error (**a**) than when the WIT stock is predicted without online news (**b**). In this case the first 35 stock data is for training the model and the rest 35 values are used for incremental learning and prediction of the next day value, making the model incrementally trainable on new incoming data, by the day.

After an integrated SNN model is trained, it is used to predict the outcome for any new input time series and on-line new samples. The regression plots in Fig. 4a,b show the original WIT values and the predicted by the model ones at a next day. The overall prediction accuracy is calculated as root mean square error at 0.06. The predictive error with the use of online news is lower than when the same index is predicted without using online news.

Using the proposed SNN-based method results in a lower predictive error when compared with the use of evolving vector-based regression techniques, such as EFuNN or DENFIS^37,50^ as shown in a comparative Supplementary Table S2.

### Predictive modelling and understanding of single stock time series and online news

This section presents positive results of integrated modelling of several temporal variables extracted from a single time series and on-line news on the predictive accuracy of one of them, along with presenting both numerical and visual analysis on the effect of news on the output variable.

#### Single stock market data

The dataset corresponds to the Wipro Limited [WIT] daily open-, high-, low-, close-, and adjusted closing prices. The observations are daily time series from January 3rd, 2017 to June 30th, 2017, with a total of 129 observations for each feature. Weekends are removed, and missing values due to holidays are replaced with the prices that came before them.

The Text News Indicator [TNI], as previously described, is used in this section to assess its impact on predicted prices and the model's usefulness. TNI is measured in the [− 1, + 1] range, with the sign indicating whether the trend is upward or downward, reflecting market sentiment. Descriptive statistics and the evolution of WIT OHLC prices are included in Suppl. Table S3 and Fig. S4.

#### Data preparation

The original dataset of the Indian Wipro Limited multinational corporation [WIT] stock is used here as a single stock data. The input variables are the temporal window sequences of five WIT stock prices: ‘open’, ‘high’, ‘low’, ‘close’, ‘adjusted close’ plus the news indicator, where the output variable is the adjusted closing price for the following day.

For comparability, this method employs the same sliding window approach as in the previous section. The original dataset is divided into 70 window samples with 5 features (no news used) and 70 samples with six features that include the news. Each sample has 60-time units (days) and one output value.

#### Results of computational modelling with the use of the proposed method

**Figure 5:**
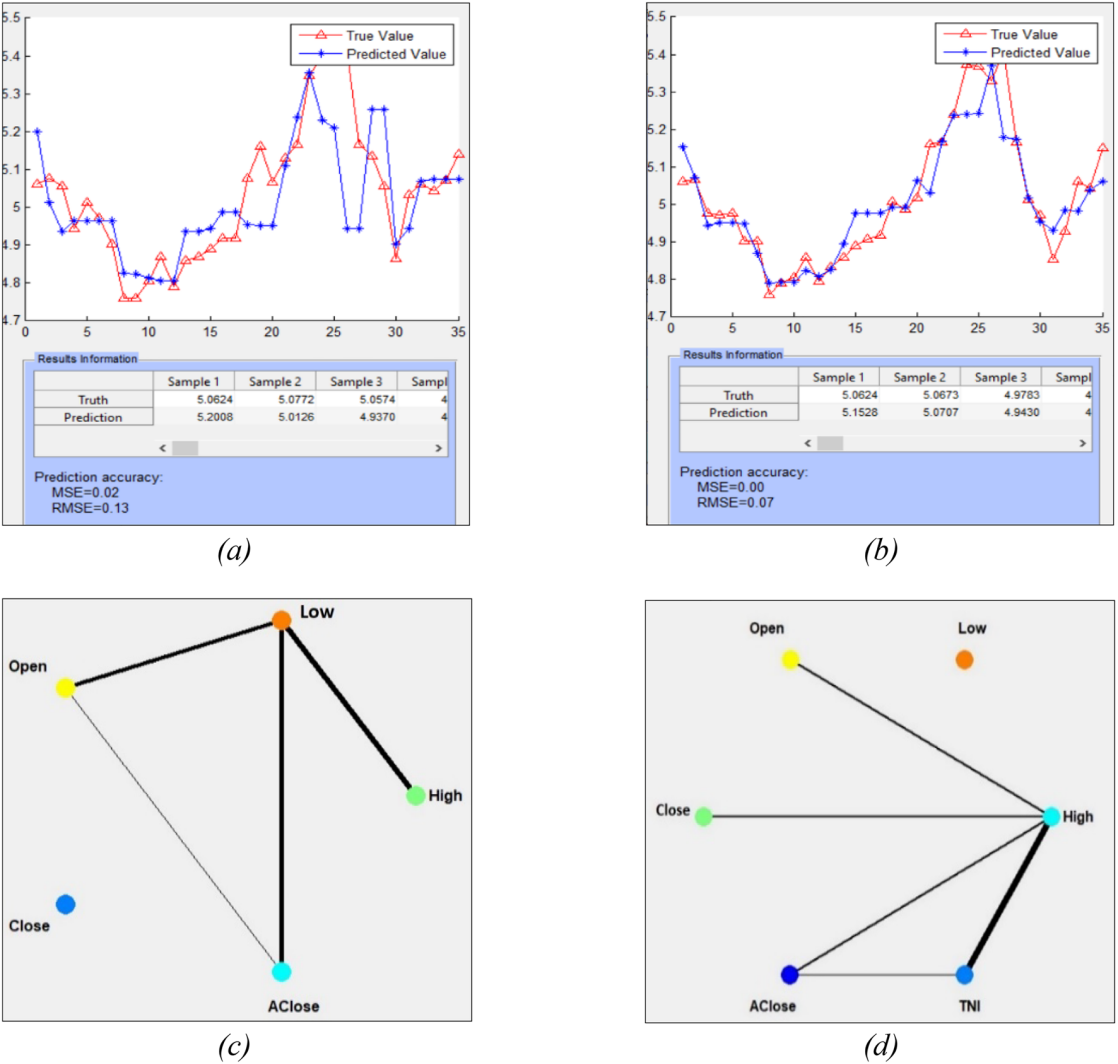
Results on predictive modelling of a single time series using its OHLC variables without- and with news information: (**a**) deSNN regression results of the adjusted closing price of WIT stock without news indicator; (**b**) with news indicator. (**c**) The variable interaction network graph between the features without news indicator, (**d**) with news indicator.

The same SNN model configuration and setting is used here as in the previous section. Modelling and analysis results with and without using news indicator are shown in Fig. 5a–d.

Figure 5a,b show that the predictive RMSE (Root mean square error) for the adjusted closing price of WIT stock after 50/50 training/testing without news is 0.13 and when using news the RMSE drops to 0.07 with. Figure 5c,d depict the level of spike interaction over time between the generated neural clusters of the five price features. The thicker the lines, the more spikes are exchanged between neural clusters during the STDP learning period in the SNN. The High price has the thickest lines in both graphs. The adjusted close price demonstrates a high level of activity with the Low and Open price without the news, indicating that more spikes were transmitted between their neurons. When news is introduced, there are broader connections between the neurons corresponding to Adjusted close, High, and TNI.

### Comparative analysis of the effect of on-line news on the predictive accuracy of each variable from a set of variables of a single stock time series, across several time series

This section presents results on integrated modelling of several temporal variables extracted from a single time series across several individual time series and on-line news, along with presenting both numerical and visual analysis on the effect of news on the output variable, being positive (decreased prediction error) or negative (increased error). The section answers the question: *Will predictive modelling of every single price from OHLC prices of a single stock and across multiple stocks benefit the same way with the inclusion of News information?*

In order to answer the above question, we have run 64 experimental models (4 × 8 × 2) on the predictive modelling of each of the 4 OHLC feature of each of the 8 stock indexes with- and without using the same TNI news indicator.

**Figure 6:**
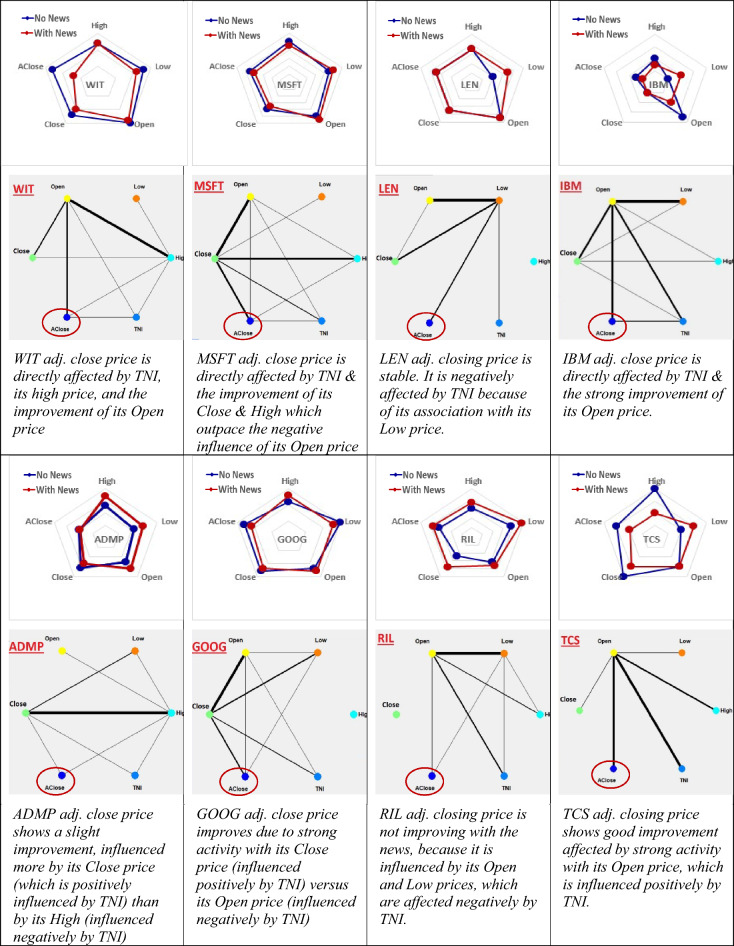
Analysis and interpretation of the results of the 64 experimental models (4 × 8 × 2) on the predictive modelling of each of the 4 OHLC features of each of the 8 stock indexes with- and without using the same TNI news indicator. For each stock index, the following explanatory information is derived to evaluate the impact of adding News information on the RMSE of the adjusted close price prediction: a radar chart, showing how close the RMSE is to zero (the center of the chart); a feature interaction network; text, explaining the impact of the encoded News as TNI index on the predictive accuracy.

In order to detect the dynamic interactions between different features of a single stock predictive model with- and without using the same TNI news index, Fig. 6 shows feature interaction network graphs for predictive models of different stocks (the adjusted closing price) with- and without TNI news. For easy comparison and analysis of model accuracy, the strengths and weaknesses of an individual model are displayed graphically on radar charts in Fig. 6. For instance, the RMSE of the WIT price models for each variable as an output with online news (shown by red dots) is closer to the center (has a smaller magnitude) than the RMSE of the model without news (the blue dots).

The numerical values of the impact on the RMSE error of the predictive SNN models on different features of individual stocks with- and without using the TNI online news index across several stocks are shown in the supplementary Table S5. Downward arrows signify a decrease in RMSE with news inclusion, upward arrows indicate an increase in RMSE, and horizontal arrows indicate no change in RMSE when news is included. These results show that while the inclusion of news can lead to a reduced predictive error of some features (as it is the case with the adjusted closing price of WIT studied above), for some other features the error increases. This is valid for other stocks as shown in Supplementary Table S5.

## Discussions

The results on multiple time series predictive modelling combined with on-line news show significantly improved predictive accuracy with the inclusion of on-line news versus no use of news. It also reveals how the time series variables interact with the on-line news variable. The overall analysis of the SNN model above (Fig. 3b) indicates that relevant news index has a normal influence on all stock indices and a stronger relationship with IBM stock. For the case study data, using news information results in positive impact on the expected accuracy for the WIT index, as it maintains its connections with MSFT while reducing its influence with LEN and TCS and replacing RIL with IBM. The maximum spikes transmitted are between LEN and ADMP, despite the fact that there is no active connection between the Adjusted close of ADMP and the News. However, it is affected indirectly by the Adjusted close prices of LEN and IBM.

The experimental results of a single time series modeling using its 4 variables (e.g., Open, High, Low and Close, abbreviated as OHLC) with or without on-line news presented in Table 1 and the related to it Fig. 8, show that on-line news can affect differently each of the 4 OHLC variables used as predictive variables. For example, on-line news decrease the prediction error when the Closed price is predicted, but for some stocks it does not favorable affecting a SNN model predicting some of the other variables as outputs. This is what we expected, but here it is revealed and explained systematically and numerically.

**Table 1:** Impact on the RMSE error of predictive SNN models of different features of individual stocks with- and without using the TNI online news index across several stocks.

RMSE	High	Low	Open	Close	Adj.Close	TNI
WIT	0.12	0.13	0.15	0.12	0.13	No
	0.12	0.11	0.14	0.10	0.07	Yes
	↔	↓	↓	↓	↓	
MSFT	0.10	0.09	0.10	0.08	0.09	No
	0.09	0.10	0.11	0.07	0.08	Yes
	↓	↑	↑	↓	↓	
LEN	0.07	0.04	0.09	0.07	0.07	No
	0.07	0.07	0.09	0.07	0.07	Yes
	↔	↑	↔	↔	↔	
IBM	0.04	0.02	0.07	0.02	0.03	No
	0.03	0.04	0.04	0.02	0.02	Yes
	↓	↑	↓	↔	↓	
ADMP	0.26	0.22	0.25	0.31	0.21	No
	0.34	0.29	0.32	0.27	0.20	Yes
	↑	↑	↑	↓	↓	
GOOG	0.11	0.15	0.12	0.13	0.13	No
	0.13	0.13	0.13	0.12	0.11	Yes
	↑	↓	↑	↓	↓	
RIL	0.15	0.19	0.16	0.12	0.16	No
	0.18	0.24	0.18	0.19	0.19	Yes
	↑	↑	↑	↑	↑	
TCS	0.04	0.02	0.03	0.04	0.03	No
	0.02	0.03	0.03	0.03	0.02	Yes
	↓	↑	↔	↓	↓	

Downward arrows signify a decrease in RMSE with news inclusion, upward arrows indicate an increase in RMSE, and horizontal arrows indicate no change in RMSE when news is included.

Through this analysis we can understand which of the time series variables, if used as predictive targets through the proposed methodology, will be positively affected by on-line news (e.g., minimizing the prediction error) and which cannot benefit from the inclusion of news information, along with the explanation for that. This can be used for the creation of better predictive systems, such as trading systems, macro-economic trends, individual health states or environmental events.

The proposed here methodology answers the main research question, namely how to understand the dynamic interaction of time series and on-line news through their integrative modelling. It offers a new method to evaluate the efficiency of using on-line news on the predictive modelling of time series.

## Conclusion

The SNN approach proposed here for the integration of times series and on-line news in predictive models, is very much inspired by the ability of the human brain to incorporate in an incremental way different temporal source of information through encoding their changes into spike sequences and then incrementally learning patterns of interactions between these sources in order to better predict future events and to gain a better understanding of the events. The SNN models capture the direction of influence between input features, so that causal associations can be identified.

Along with numerical estimation of the impact of news on the predictive accuracy of time series variables, the method offers an interpretation and explanation.

Many questions still remain for further studies, such as:

- the choice of news sources;
- how their relevance to the predicted outputs changes over time;
- parameter optimization of the SNN models for better performance;
- a better integration of human knowledge and machine intelligence.

With the fast development of the massively parallel and low energy neuromorphic and quantum hardware platforms, that are complementing and substituting the traditional von Neuman machines^30,31^, now the challenge for machine intelligence is to develop suitable for this hardware and also efficient and explainable computational models, such as the proposed SNN approach^40–42^.

The proposed methodology, being a generic one, can be extended to specific methods for different domain areas (along with the illustrated financial area)^31,44–48^:

- cognitive studies, where EEG and fMRI data, that are of different spatial and temporal resolutions, can be integrated on the same time scale for a SNN model to be trained;
- seismic studies for earthquake prediction, where seismic time series data can be integrated with GPS and satellite image time series data);
- personalized wearable devices for personalized health, where ECG data can be integrated with other streaming personal information, such as physical activity, nutrition, sleep, etc.
- global warming prediction on different time scales, where relevant multiple and multimodal streaming information is integrated, including: temperature, air pollution, volcanic activities, solar eruptions, human population, green and fossil energy, etc.

The team is working on the implementation of the proposed model on the latest neuromorphic platforms to make this method available across application areas.

## Methods

**Figure 7:**
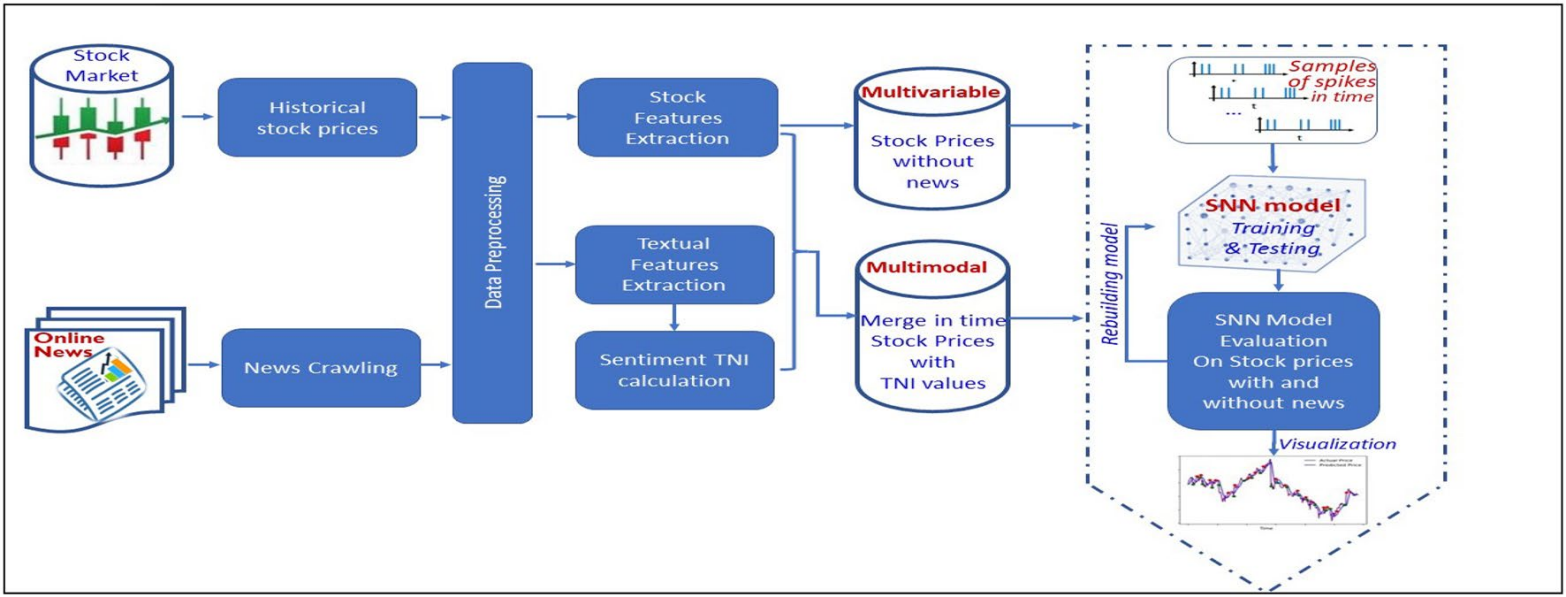
A functional diagram of the prosed SNN-based methodology illustrated on the case study of financial stock data.

The proposed here methodology consists of three groups of methods as illustrated in Fig. 7 on the case study of financial stock data modelling:

1. Feature extraction and encoding of all streaming data and information as uniformly integrated numerical multimodal time series on the same time scale;
2. Predictive modelling of the integrated multimodal times series in a SNN;
3. Discovery of explainable dynamic temporal patterns of association between multimodal time series variables, including news time series.

### Encoding news as time series

The main idea here is that all streaming data and information is first uniformly represented as numerical time series on the same time scale, forming a set of multimodal and uniformly represented time series, using the same time unit of measurement, e.g., seconds, days, years, etc. For financial time series and online news, the time unit would be a day.

Here we propose how online news can be encoded into a numerical time series. A Turning News Index (TNI) is proposed in this paper to mitigate data-driven bias in news selection. The TNI index is linked in our case study to stock prices and is calculated using the 'Bag of Words' (BoW) method.

A ‘Bag of words’ is a traditional feature extraction approach in which each word is represented as a feature. BoW is a simple approach that measures the importance of words based on their frequency in the document while ignoring word order. As a result, News is classified as 'direct' or 'indirect' based on the occurrence of the word and the topic of the news. For instance, any news referring to a specific topic, such as Wipro stock news, will have a direct impact on the prediction and will thus be classified as 'direct.' In the case of indirect news, its impact is on the overall trend of the stock market rather than on a specific stock. And will be labelled as 'indirect.'

In this paper, the classified news is quantified using the Bag of Words (BoW) approach, and the n-gram model with TF-IDF is used to calculate TNI (Eqs. 1, 2, 3, 4).

1 $$TNI=\left\{\mathrm{BoW},\mathrm{n}-\mathrm{gram}\right\}; \mathrm{TNI}\in [-\mathrm{1,1}]$$

2 $$tf(t,d)=\frac{{f}_{t,d}}{\sum_{{t}{^\prime}\in d}{f}_{{t}{^\prime},d}}$$

3 $$idf\left(t,D\right)=log\frac{N}{|\{d\in D:t\in d\}|}$$

4 $$tfidf\left(t,d,D\right)=tf\left(t,d\right)\cdot idf(t,D)$$

where *tf* is the term frequency, which determines the significance of a term in a document. It computes the frequency of occurrence of a term 't' in one document 'd' with respect to the total number of terms in the same document 'd'; *idf* is the Inverse Document Frequency, which measures the rarity of a term. The *idf* of a term 't' is the log of the ratio of the total number of documents 'N' in the domain set to the number of documents 'D' in which that term appears; thus, *tfidf* assigns a numerical weight to words based on how important a word is to a corpus, or collection of documents.

The upward and downward trends are analyzed using numerical values from the input text rather than just sentimental values. The news can be quantified in the [− 1, + 1] range, where the sign indicates whether the trend is upward or downward, and in the [0, 1] range to indicate the probability value of news impact on the stock. Supplementary Table S4 shows a sample of news classifications and TNI values, while Supplementary Fig. S5 displays selected online news as a time series.

### Integrating news TNI and time series data into a multimodal time series

We assume that there is a set of *n* continuous-value time series TS = {TS1, TS2, …, TSn}, one of them denoted here as TSo being a target for predicting its next time value. For the case study, a TNI index series is also created as explained above. At any time, t_i_, an input vector is created (Eq. 5).

5 $${\text{V}}\left( {{\text{t}}_{{\text{i}}} } \right) = \left\{ {{\text{TS1}}\left( {{\text{t}}_{{\text{i}}} } \right),{\text{TS2}}\left( {{\text{t}}_{{\text{i}}} } \right), \ldots ,{\text{TSn}}\left( {{\text{t}}_{{\text{i}}} } \right),{\text{TNI}}\left( {{\text{t}}_{{\text{i}}} } \right)} \right\},$$

A moving window of time T = {t_1_, …, t_l_} is used to incrementally train a SNN model to predict TSo (t_l+1_) when using the sequence of all vectors V((t_1_), …,V(t_l_)) that form a training multivariate temporal sample S (t_1−_t_l_) (Eq. 6).

6 $${\text{S}}\left( {{\text{t}}_{{{1} - }} {\text{t}}_{{\text{l}}} } \right) = \left\{ {{\text{V}}\left( {{\text{t}}_{{1}} } \right),{\text{V}}\left( {{\text{t}}_{{2}} } \right), \ldots ,{\text{V}}\left( {{\text{t}}_{{\text{l}}} } \right)} \right\}$$

### Encoding of the multimodal times series into spike trains

**Figure 8:**
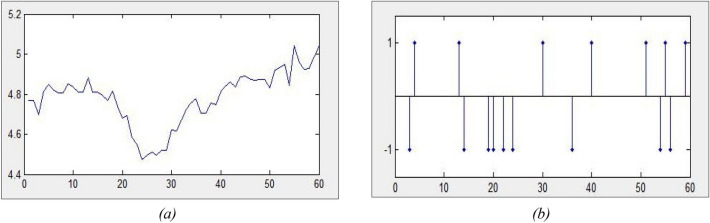
Encoding time series into spike sequences: (**a**) exemplar, continuous value time series data; (**b**) the same time series, encoded into a spike sequence, reflecting on the changes in the data over consecutive times.

First, the real-value input data is transformed from continuous values to discrete sequences of spikes, each spike used to encode a change in the data over consecutive time moments, exemplified in Fig. 8a,b. The rationale behind this is that we are interested to capture dynamic changes of the data over time in relation to the TNI online news and between each other. These changes can represent predictive and informative patterns.

Different methods, such as the threshold-based representation encoding method can be used, with a threshold, to discretize the continuous streams of time series data into spikes^31,32^. This method is based on thresholding the difference between two consecutive values of the same input variable over time. As a result, it generates a positive spike that encodes an increased value at the next time point, and a negative spike that encodes a decreased value at the next time point. The spike information representation simplifies the input data by focusing on the changes in the data over time rather than using their real values in the followed modelling procedure that aims at learning dynamic patterns of these changes that relate and trigger certain outcomes (Fig. 8a,b). Each input variable generates both positive and negative spikes that are entered in the SNNcube to create spiking activity across other neurons in the model.

### Training a SNN model on the integrated encoded multimodal time series and news

Our choice of a SNN for predictive and explainable multimodal data modelling is the NeuCube architecture^32^. The reason is that NeuCube includes both a 3D SNNcube, that can be trained on streaming data to capture spatio-temporal patterns, and a deSNN classifier/regressor^38^ that is trained to predict an output variable based on the learned in the Cube dynamic spatio-temporal patterns. A description of the NeuCube functionality is given in the Supplementary material, Fig. S6^49^.

The input variables, including the news variable, are mapped into the 3D SNNcube based on their time series similarity, so that similar input features are allocated to closer spiking neurons (input neurons) in the 3D space^43,50^. As a result, the transformed input data into spike trains are mapped into spatially located neurons in the SNNcube, with a defined spike rate of and time length. The encoded spike sequences are continuously fed into the SNNcube reservoir in the order in which they were encoded.

The SNNcube is trained incrementally in an unsupervised mode using the STDP rule on the samples S of the integrated time series and online news data. The STDP learning rule considers the time of spiking between two connected neurons, so that consecutive changes in data from one time to another across all input variables are learned. STDP is expressed in terms of STDP learning window $({\tau }_{pre}-{\tau }_{post})$ in which the difference between the time of a pre-synaptic spike and the time of a post-synaptic spike will determine the synaptic weight change dW (Eq. 7). In the equation,${\tau }_{+}$ and ${\tau }_{-}$ refer to the time of spikes at the pre-synaptic and post-synaptic neurons in the SNNcube; A_+_ and A_−_ refer to the maximum fraction of synaptic adjustment if ${\tau }_{pre}<{\tau }_{post}$ approaches to zero.

7 $$dW\left({\tau }_{pre}-{\tau }_{post}\right)= \left\{\begin{array}{c}{A}_{+ }\mathrm{exp}\left(\frac{{\tau }_{pre}-{\tau }_{post}}{{\tau }_{+}}\right) if {\tau }_{pre}<{\tau }_{post}\\ {A}_{- }\mathit{exp}\left(-\frac{{\tau }_{pre}-{\tau }_{post}}{{\tau }_{-}}\right) if{ \tau }_{pre}>{\tau }_{post}\end{array}\right.$$

After training on input samples S (t_1_ − t_l_), an output deSNN regressor is trained again on the same data to associate the spiking activity of the SNNcube for every input sample S (t_1−_t_l_) = {V(t_1_), V(t_2_), …, V(t_l_)} with its corresponding desired output value of TSo (T_l+1_) (Eqs. 8, 9).

8 $$\Delta {w}_{j,i}={mod}^{order\left({a}_{j}\right)}$$

9 $$\Delta {w}_{j,i}\left(t\right)={e}_{j}\left(t\right)\cdot D$$

where w_j,i_ is the weight between neuron j of the SNNcube and neuron i in the deSNN output regressor; mod ∈ (0,1) is the modulation factor used to establish the initial value of the wight w_j,i_; order(a_j_) is the order of arrival of a first spike from neuron j to neuron i at each input to neuron i; e_j_(t) = 1 if there is a consecutive spike at neuron j at time t during the presentation of the input sample S(t_1−_t_l_); e_j_(t) = − 1otherwise, indicating that there is no spike at time t at neuron j to be transferred to neuron i; D is a drift parameter, which can be different for ‘spike’ or ‘no spike’ drifts.

In the experiments above the following parameter values of the NeuCube model are used. A threshold-based encoding method with 0.5 spike rate is adopted. The SNNcube unsupervised learning parameters Leaking rate, STDP rate, firing threshold, training rounds, refractory time, and long-distance probability are respectively set to 0.0002; 0.1; 0.5; 1; 6; and 3. The deSNNs regressor’s supervised learning parameters modulation factor (mod), drift adjustments (D); number of nearest neurons, and sigma are set respectively to 0.5, 0.005, 3 and 1 (see for details^31,49^).

### Extracting explainable information from the model

During the unsupervised incremental learning of multiple input samples S (t1 − tl) of the integrated time series, neurons in the SNNcube are clustered based on their connections to input neurons. In addition, dynamic information exchange is captured in a dynamic variable interaction graph as illustrated in Figs. 3, 5 and 6. The thicker the line between two input variables, the stronger the temporal relationship between them, meaning that changes in one variable (represented as a spike) relates to a change of the other variable at the next time (a spike at the next time).

More details of the proposed in this section SNN-based method for predictive modelling and understanding of the dynamic interaction between all input variables (e.g., time series variables and online news) are explained and illustrated in the Supplementary material.

### Supplementary Information

Supplementary Information.

## Data Availability

The data sets that are used to support the proposed methodology and the findings of this study are available from Share Market Data {https://www.kaggle.com/itoeiji/deep-reinforcement-learning-on-stock-data}. Encoded time series news data for the SNN models are available from the authors upon request. The system development software NeuCube^49^ and NeuCom^50^, used for the creation of the integrated times series and on-line news experimental models, are available free to use.
