# Supplementary material for: Spiking neural networks for predictive and explainable modelling of multimodal streaming data with a case study on financial time series and online news
Source: Sci Rep. 2023 Oct 26;13:18367. doi: 10.1038/s41598-023-42605-0 (PMC10603166; doi:10.1038/s41598-023-42605-0)
Supplement: Supplementary file 1 — Supplementary Information. [file 41598_2023_42605_MOESM1_ESM.docx]

**Spiking Neural Networks for Predictive and Explainable Modelling of Multimodal Streaming Data with a Case Study on Financial Time-series and Online News**

**<< Supplementary Material >>**

Iman AbouHassan*^1,2^, Nikola K. Kasabov* ^3,4,5^, Vinayak Jagtap ^6^, Parag Kulkarni ^6,7^

1. Technical University of Sofia, Bulgaria.
2. Central Bank of Lebanon, Lebanon.
3. KEDRI, SECMS, Auckland University of Technology, New Zealand.
4. Ulster University, the UK.
5. IICT, Bulgarian Academy of Sciences, Sofia, Bulgaria.
6. College of Engineering, Pune, India.
7. Tokyo International University, Japan.

* Contact persons: Iman AbouHassan ([iabouhassan@tu-sofia.bg](mailto:iabouhassan@tu-sofia.bg) and [iabouhassan@bdl.gov.lb](mailto:iabouhassan@bdl.gov.lb)) and Nikola K. Kasabov ([nkasabov@aut.ac.nz](mailto:nkasabov@aut.ac.nz)),

|  | *WIT* | *MSFT* | *LEN* | *IBM* | *ADMP* | *GOOG* | *RIL* | *TCS* |  | *TNI* |
| --- | --- | --- | --- | --- | --- | --- | --- | --- | --- | --- |
| *Mean* | *4.92* | *62.61* | *47.94* | *140.30* | *3.94* | *868.06* | *608.81* | *1,106.42* |  | *-0.17* |
| *Standard Error* | *0.02* | *0.25* | *0.27* | *0.74* | *0.06* | *5.41* | *5.92* | *4.14* |  | *0.01* |
| *Median* | *4.89* | *61.67* | *49.16* | *141.34* | *3.85* | *835.67* | *632.02* | *1,108.06* |  | *-0.18* |
| *Standard Deviation* | *0.21* | *2.86* | *3.03* | *8.45* | *0.63* | *61.47* | *67.22* | *47.03* |  | *0.10* |
| *Sample Variance* | *0.04* | *8.17* | *9.19* | *71.38* | *0.40* | *3,778.66* | *4,518.00* | *2,211.37* |  | *0.01* |
| *Kurtosis* | *0.26* | *-1.04* | *-0.89* | *-1.54* | *-0.51* | *-1.31* | *-1.15* | *-0.53* |  | *1.06* |
| *Skewness* | *0.37* | *0.42* | *-0.76* | *-0.06* | *0.47* | *0.56* | *-0.61* | *0.14* |  | *0.60* |
| *Minimum* | *4.47* | *58.41* | *41.41* | *127.80* | *3.00* | *786.14* | *491.87* | *996.04* |  | *-0.39* |
| *Maximum* | *5.42* | *68.80* | *52.12* | *153.14* | *5.75* | *983.68* | *696.49* | *1,238.68* |  | *0.19* |

Table S1: Descriptive Statistics of the used financial stock time series data

Fig. S1: Evolution of selected stock prices (Jan-Jun 2017, in USD).


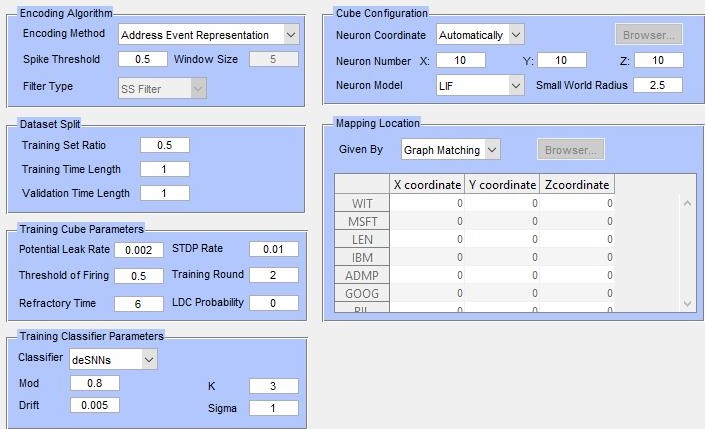


Fig. S2: Our SNN model parameter setting panel

| Model/Method | News indicator | Training/Test mode | RMSE |
| --- | --- | --- | --- |
| Our SNN model | No | 50/50 | 0.08 |
| Our SNN model | Yes | 50/50 | 0.06 |
| Our SNN model | Yes | 3-K fold | 0.05 |
| NeuCom/EFuNN | Yes | 50/50 | 0.43 |
| NeuCom/EFuNN | Yes | 80/20 | 0.24 |
| NeuCom/DENFIS | Yes | 50/50 | 0.53 |
| NeuCom/DENFIS | Yes | 80/20 | 0.17 |

*Table S2: Comparative results of Wipro Limited [WIT] stock prediction price when using the proposed method versus the use of traditional vector-based evolving connectionist systems EFuNN and DENFIS from the NeuCom environment [32, 40].*

Supplementary Fig. S3 (a) shows the interactions of time-series variables when our SNN model is created to predict WIT stock without using online news. In the absence of news indicator, the analyzed feature [WIT] is heavily influencing/influenced by [TSC] and [LEN], but less so by [MSFT] and [RIL]. It does, however, show a high correlation with other indices. The impact of the time-series variables on the whole model is visualized in Supplementary Fig. S3 (b). The proportion of neurons in the NeuCube that are part of an input neuron cluster ranges from 3% for the [GOOG] index to 21% for the [RIL] index. The examined feature [WIT] accounts for 8% of the total. This type of analysis allows to compare directly the temporal association between the input variables in a SNN model that is using news versus a SNN model that does not use news.

| 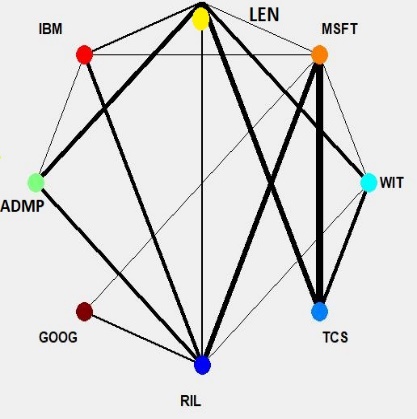(a) | 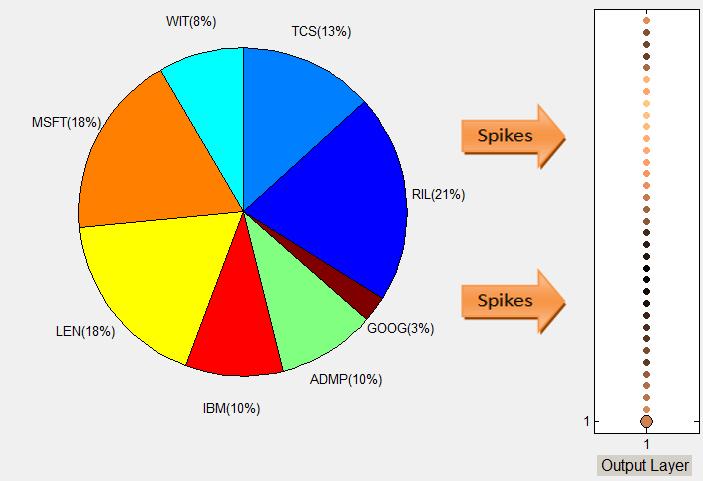(b) |
| --- | --- |
| Fig. S3 (a) The variable interaction network of a SNN predictive model to predict WIT stock without using online news; (b) The proportion of spiking neurons connected (influenced) by the corresponding input time-series variables. | |

| ***Wipro Limited [WIT]*** | ***Open*** | ***High*** | ***Low*** | ***Close*** | ***Adj.Close*** | ***TNI*** |
| --- | --- | --- | --- | --- | --- | --- |
| *Mean* | *4.98* | *5.02* | *4.94* | *4.98* | *4.92* | *-0.17* |
| *Standard Error* | *0.02* | *0.02* | *0.02* | *0.02* | *0.02* | *0.01* |
| *Median* | *4.95* | *4.99* | *4.89* | *4.95* | *4.89* | *-0.18* |
| *Standard Deviation* | *0.21* | *0.21* | *0.20* | *0.21* | *0.21* | *0.10* |
| *Sample Variance* | *0.04* | *0.04* | *0.04* | *0.04* | *0.04* | *0.01* |
| *Kurtosis* | *0.50* | *0.35* | *0.32* | *0.32* | *0.26* | *0.96* |
| *Skewness* | *0.48* | *0.38* | *0.40* | *0.37* | *0.37* | *0.59* |
| *Minimum* | *4.53* | *4.58* | *4.50* | *4.53* | *4.47* | *-0.39* |
| *Maximum* | *5.55* | *5.55* | *5.45* | *5.48* | *5.42* | *0.19* |

Table S3: Descriptive Statistics of the WIT stock

Fig. S4: Behavior of WIT stock features.

| **News** | **News Link** | **Class** | **TNI** |
| --- | --- | --- | --- |
| 1. Production outages in Asia lend slight support to prices | http://feeds.reuters.com /~r/reuters/ INbusinessNews /~3/NREDmlBQ6wc/global-lng-idINKBN19L14Q | Indirect | 0.032 |
| 1. UK consumers suffer the longest decline in spending power since the 1970 s | http://feeds.reuters.com/~r/reuters/ INbusinessNews /~3/Xsx0vFoiNeg/britain-economy-idINKBN19L144 | Indirect | -0.09558 |
| 1. Market Now: Gammon Infra, GVK Power Infra surge over 10% | http://economictimes.indiatimes.com/ markets/ stocks/news/market-now-gammon-infra-gvk-power-infra-surge-over-10/articleshow/59384964.cms | Direct | -0.13671 |
| 1. Global markets: Brightening economy sets euro up for strongest quarter since debt crisis | http://feeds.reuters.com /~r/reuters/ INbusinessNews/~3/jAAVKhzW9jo/global-markets-idINKBN19L13U | Indirect | -0.04412 |

Supplementary Table S4: Selected News Topics, Links, and TNI calculation.

Supplementary Fig. S5: Turning News Index (TNI).

**The NeuCube SNN Architecture and Functionality (A brief description)**

The proposed in the paper model uses in one of its modules the brain-inspired NeuCube SNN architecture [27]. Its learning and classification algorithms are presented in [27,26] and a software implementation is available [39].

The NeuCube architecture consists of three modules: (a) input data encoding module; (b) 3D SNNcube module, where unsupervised training adjusts the initial connection weights using the STDP learning rule [30]; and (c) output deSNN regression/classification module for supervised training, that associates the output information with each trained spatio-temporal sample after the sample is propagated through the SNNcube [33].


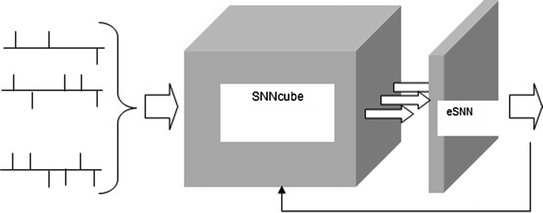


(a) (b) (c)

Fig.S6. A functional diagram of the NeuCube SNN architecture: (a) The input data encoding module, which transforms continuous input data into trains of spikes, uses encoding algorithms, such as the Ben's Spiker Algorithm (BSA), Temporal Contrast (Threshold based), Step Forward Spike Encoding Algorithm (SF), or Moving Window Spike Encoding Algorithm (MW) [26]; (b) 3D SNN cube module, where spatio-temporal data are mapped into predefined areas and unsupervised training of STDP is applied, that allows learning spatio-temporal relations from input data; (c) Output deSNN regression/ classification module for supervised learning, where a new output neuron is dynamically allocated and connected to the neurons in the SNNcube using Rank Order rule and a drift parameter. Neurons with similar weight vectors are merged using Euclidean distance.

The overall accuracy of a NeuCube model depends on learning parameters in the SNNcube and the regressor/classifier deSNN. During learning, the SNNcube creates/modifies connections between neurons based on their spiking activity in time, so that further analysis of a trained SNNcube can reveal dynamic temporal patterns of interaction between input variables. And that is why NeuCube is used in the next section for the development of a generic method for online, predictive time series modelling together with online news and for the explanation of their dynamic relationship.

| **RMSE** | **High** | **Low** | **Open** | **Close** | **Adj.Close** | **TNI** |
| --- | --- | --- | --- | --- | --- | --- |
| **WIT** | 0.12 | 0.13 | 0.15 | 0.12 | 0.13 | No |
|  | 0.12 | 0.11 | 0.14 | 0.10 | 0.07 | Yes |
|  | ↔ | ↓ | ↓ | ↓ | ↓ |  |
| **MSFT** | 0.10 | 0.09 | 0.10 | 0.08 | 0.09 | No |
|  | 0.09 | 0.10 | 0.11 | 0.07 | 0.08 | Yes |
|  | ↓ | ↑ | ↑ | ↓ | ↓ |  |
| **LEN** | 0.07 | 0.04 | 0.09 | 0.07 | 0.07 | No |
|  | 0.07 | 0.07 | 0.09 | 0.07 | 0.07 | Yes |
|  | ↔ | ↑ | ↔ | ↔ | ↔ |  |
| **IBM** | 0.04 | 0.02 | 0.07 | 0.02 | 0.03 | No |
|  | 0.03 | 0.04 | 0.04 | 0.02 | 0.02 | Yes |
|  | ↓ | ↑ | ↓ | ↔ | ↓ |  |
| **ADMP** | 0.26 | 0.22 | 0.25 | 0.31 | 0.21 | No |
|  | 0.34 | 0.29 | 0.32 | 0.27 | 0.20 | Yes |
|  | ↑ | ↑ | ↑ | ↓ | ↓ |  |
| **GOOG** | 0.11 | 0.15 | 0.12 | 0.13 | 0.13 | No |
|  | 0.13 | 0.13 | 0.13 | 0.12 | 0.11 | Yes |
|  | ↑ | ↓ | ↑ | ↓ | ↓ |  |
| **RIL** | 0.15 | 0.19 | 0.16 | 0.12 | 0.16 | No |
|  | 0.18 | 0.24 | 0.18 | 0.19 | 0.19 | Yes |
|  | ↑ | ↑ | ↑ | ↑ | ↑ |  |
| **TCS** | 0.04 | 0.02 | 0.03 | 0.04 | 0.03 | No |
|  | 0.02 | 0.03 | 0.03 | 0.03 | 0.02 | Yes |
|  | ↓ | ↑ | ↔ | ↓ | ↓ |  |

*Table S5. Analysis and interpretation of the results of the 64 experimental models (4 x 8 x2 ) on the predictive modelling of each of the 4 OHLC features of each of the 8 stock indexes with- and without using the same TNI news indicator. The table shows in a numerical form the impact on the RMSE error of predictive SNN models of different features of individual stocks with- and without using the TNI online news index across stocks. Downward arrows signify a decrease in RMSE with news inclusion, upward arrows indicate an increase in RMSE, and horizontal arrows indicate no change in RMSE when news is included.*
